# Diverse roles of endoplasmic reticulum stress sensors in bacterial infection

**DOI:** 10.1186/s40348-016-0037-7

**Published:** 2016-02-16

**Authors:** Helena Pillich, Maria Loose, Klaus-Peter Zimmer, Trinad Chakraborty

**Affiliations:** Institute of Medical Microbiology, Justus-Liebig-University Giessen, 35392 Giessen, Germany; Department of Pediatrics, Justus-Liebig-University Giessen, 35392 Giessen, Germany

**Keywords:** Endoplasmic reticulum stress, Bacterial infection, Bacterial effectors

## Abstract

Bacterial infection often leads to cellular damage, primarily marked by loss of cellular integrity and cell death. However, in recent years, it is being increasingly recognized that, in individual cells, there are graded responses collectively termed cell-autonomous defense mechanisms that induce cellular processes designed to limit cell damage, enable repair, and eliminate bacteria. Many of these responses are triggered not by detection of a particular bacterial effector or ligand but rather by their effects on key cellular processes and changes in homeostasis induced by microbial effectors when recognized. These in turn lead to a decrease in essential cellular functions such as protein translation or mitochondrial respiration and the induction of innate immune responses that may be specific to the cellular deficit induced. These processes are often associated with specific cell compartments, e.g., the endoplasmic reticulum (ER). Under non-infection conditions, these systems are generally involved in sensing cellular stress and in inducing and orchestrating the subsequent cellular response. Thus, perturbations of ER homeostasis result in accumulation of unfolded proteins which are detected by ER stress sensors in order to restore the normal condition. The ER is also important during bacterial infection, and bacterial effectors that activate the ER stress sensors have been discovered. Increasing evidence now indicate that bacteria have evolved strategies to differentially activate different arms of ER stress sensors resulting in specific host cell response. In this review, we will describe the mechanisms used by bacteria to activate the ER stress sensors and discuss their role during infection.

## Introduction

Newly synthesized transmembrane and secretory proteins are folded and post-translationally modified within the endoplasmic reticulum (ER). Certain conditions such as hypoxia, Ca^2+^ perturbation, and reactive oxygen species (ROS) cause continued accumulation of unfolded proteins within the ER, a condition termed ER stress. To counteract ER stress and maintain ER function, cells activate the unfolded protein response (UPR), a signaling cascade composed of three axes which enables the reduction of protein amount entering the ER by translational inhibition, enhancement of protein folding by transcriptional upregulation of ER chaperones, and degradation of misfolded proteins through ER-associated degradation (ERAD). If ER stress is prolonged and severe, UPR induces apoptosis.

Several studies have now implicated UPR in bacterial infections. Surprisingly, it becomes clear that some bacteria have evolved strategies to activate all three UPR-signaling pathways. Recent studies have revealed that the execution of a particular UPR-signaling pathway does not occur randomly and new functions of the ER stress sensors have been described. In this review, we summarize the mechanisms used by bacteria to induce UPR and discuss the importance of certain UPR-signaling pathway activation.

## UPR-signaling pathways

In higher eukaryotes, UPR signaling is mediated by the ER-transmembrane proteins detecting ER stress: inositol-requiring enzyme 1 (IRE1), protein kinase RNA (PKR)-like ER kinase (PERK), and activating transcription factor 6 (ATF6). The ER-resident chaperone immunoglobulin heavy chain-binding protein (BiP) binds to the luminal domain of the ER stress sensors and keeps them in an inactive state. Accumulation of unfolded proteins leads to release of BiP from the ER stress sensors and subsequent binding to the unfolded proteins [1].

**Fig. 1:**
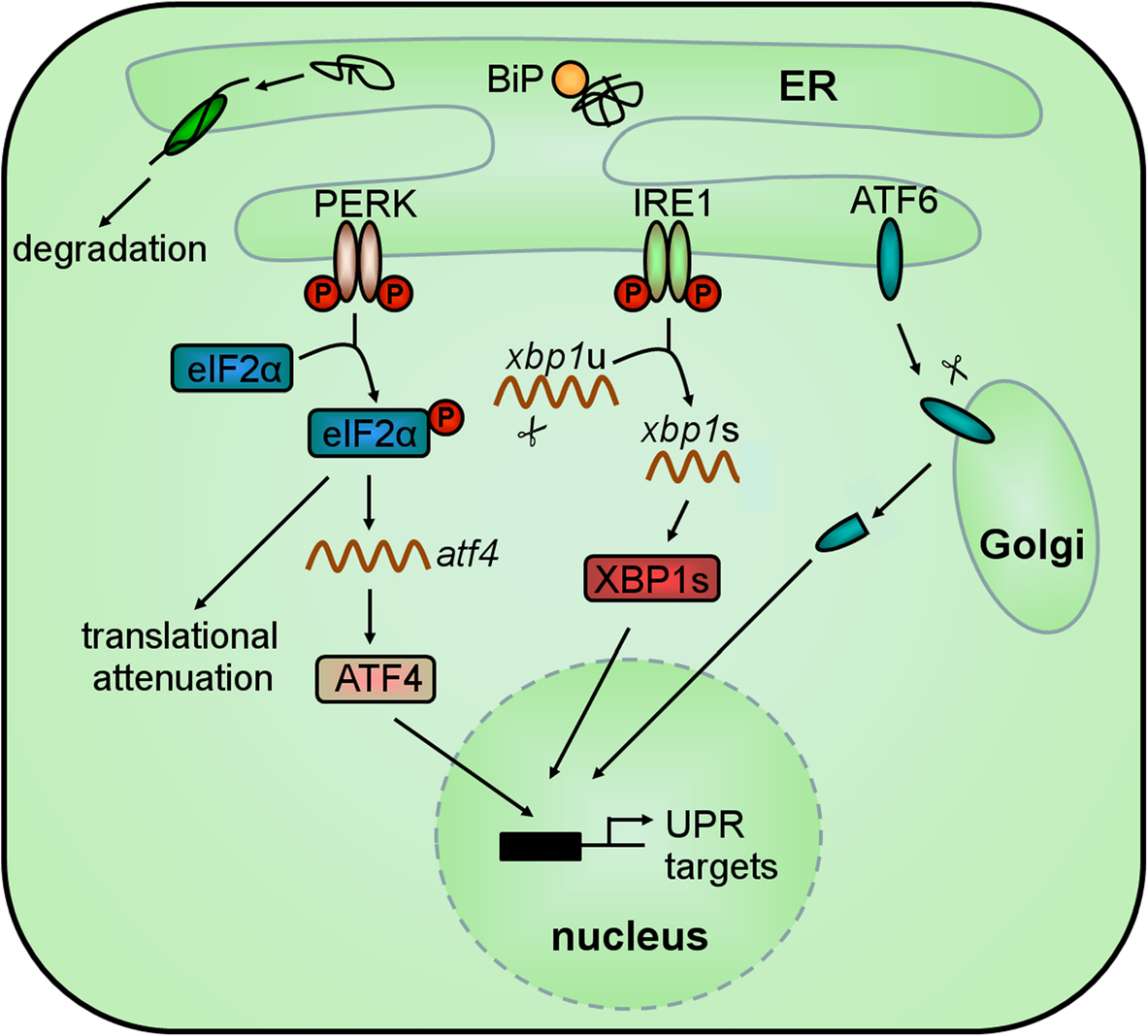
The UPR-signaling pathway. Proteins that are not properly folded within the ER are retro-translocated into the cytoplasm for degradation using the ERAD mechanism. Under ER stress conditions, unfolded proteins accumulate within the ER leading to dissociation of BiP from the ER stress sensors IRE1, PERK, and ATF6. This leads to oligomerization and autophosphorylation of IRE1 and PERK. Active IRE1 splices the *xbp1* mRNA producing the spliced XBP1. Active PERK acts as a kinase of eIF2α. Under this condition, the global translation is attenuated. Thus, the protein amount entering the ER is reduced. However, the translation of *atf4* mRNA is efficiently increased. Release of BiP from ATF6 permits the translocation of ATF6 to the Golgi apparatus where it is cleaved by two proteases. The resulting cytosolic portion of ATF6, ATF4, and spliced XBP1 enter the nucleus and functions as transcription factors of UPR target genes

Unbound IRE1 oligomerizes, autophosphorylates, and activates its endoribonuclease which mediates unconventional splicing of an intron from X-box-binding protein 1 (*xbp1*) messenger RNA (mRNA) producing a potent transcription factor (spliced-XBP1) [1] (Fig. 1).

Disruption of the PERK-BiP complex results in dimerization, autophosphorylation and activation of the kinase domain of PERK. Active PERK phosphorylates serine 51 of eukaryotic translation initiation factor 2α (eIF2α) consequently leading to translational inhibition. Thus, the protein load that enters the ER decreases. However, under this condition, the translation of some mRNAs, such as that of the transcription factor *atf4*, is increased [1] (Fig. 1).

Release of BiP from ATF6 permits the translocation of ATF6 to the Golgi apparatus where it is cleaved by two proteases. The cytosolic fragment migrates to the nucleus and regulates the transcription of UPR target genes [1] (Fig. 1).

## Bacterial mechanisms to induce UPR

**Fig. 2:**
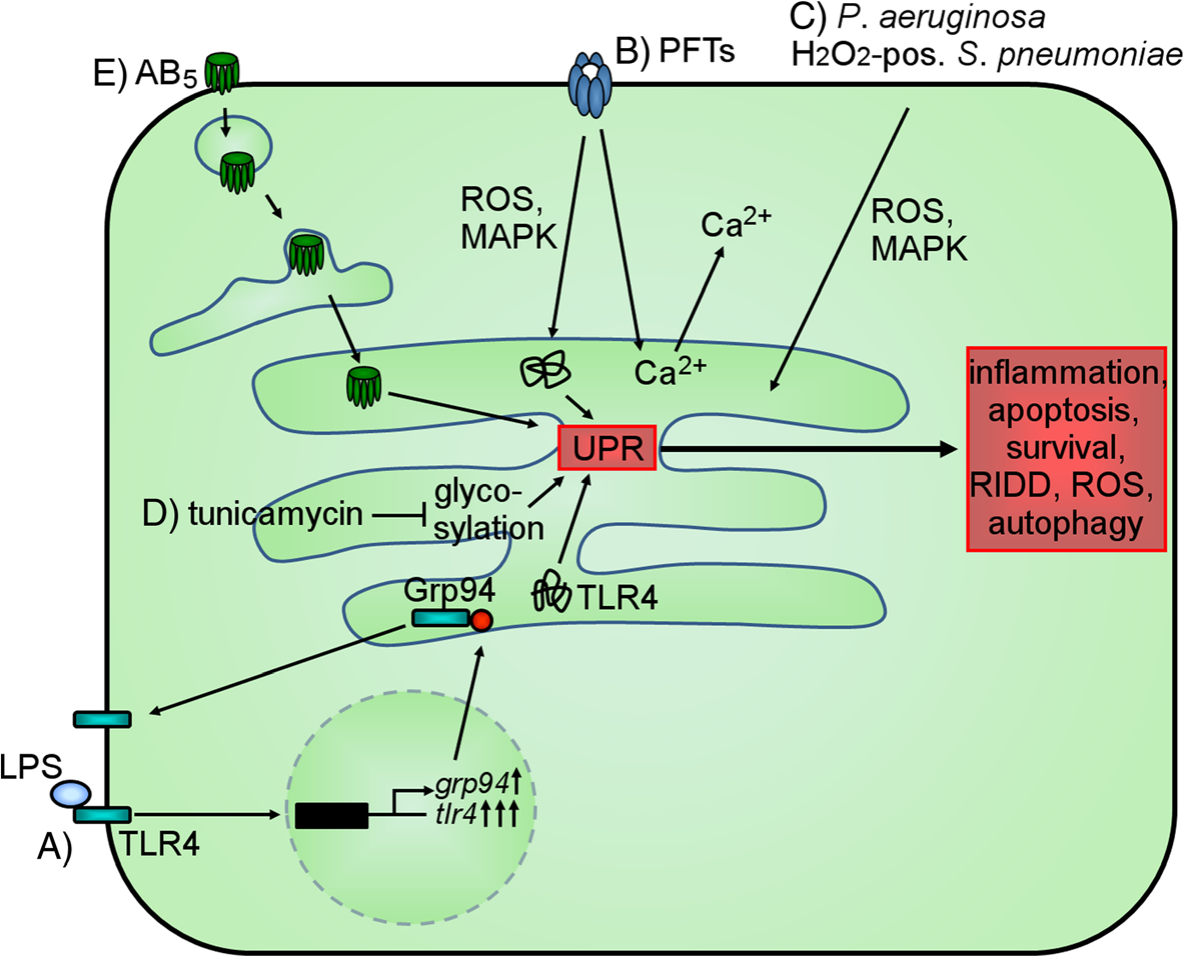
Mechanisms of bacteria inducing UPR. (*A*) Grp94 chaperones TLR4 which is activated by binding of LPS. Under LPS stimulus, TLR4 is endocytosed and its expression is increased. In addition, the expression of *grp94* is increased with a much lower magnitude than that of *tlr4* resulting in accumulation of unfolded TLR4 within the ER. (*B*) PFTs induce ROS production, MAPK activation, and Ca^2+^ influx as well as induction of ER Ca^2+^ release resulting in UPR activation. (*C*) *P. aeruginosa* and bacterial-produced H_2_O_2_ induce UPR by MAPK activation and increase of ROS. (*D*) Tunicamycin inhibits N-glycosylation of proteins. (*E*) AB_5_ toxins are endocytosed and transported via the Golgi apparatus to the ER where they induce UPR activation because they are unfolded, cleave BiP, or interact with IRE1

Bacteria have evolved different virulence factors that trigger the activation of UPR. One well-known example is lipopolysaccharide (LPS), an endotoxin located in the outer membrane of Gram-negative bacteria. LPS is detected by toll-like receptor 4 (TLR4) that is delivered by the ER chaperone heat shock protein 90 kDa beta member 1 (Grp94) from the ER to the plasma membrane. Expression of TLR4 and Grp94 is increased after LPS treatment. However, folding and plasma membrane translocation of TLR4 is not sufficient because the expression level of Grp94 is lower than that of TLR4 [2] (Fig. 2 (A)).

Another major group of toxins that induce UPR are pore-forming toxins (PFT) including aerolysin, Cry5B, listeriolysin O (LLO), and the small protein early secretory antigenic target 6 (ESAT-6) [3–5]. PFT-mediated pore formation on the plasma membrane which leads to perturbance of Ca^2+^ levels, activation of mitogen-activated protein kinase (MAPK), and induction of ROS production triggers UPR activation [3, 4] (Fig. 2 (B)). These circumstances are also important for UPR-activation mechanisms utilized by other bacteria, for instance *Pseudomonas aeruginosa* and H_2_O_2_-positive *Streptococcus pneumoniae* [6, 7] (Fig. 2 (C)).

Other bacteria have evolved strategies to activate UPR by release of factors that are able to enter the lumen of the ER. This includes tunicamycin, an inhibitor of protein N-linked glycosylation, which is very often used as a positive control for UPR induction [5, 7, 8] (Fig. 2 (D)). Also, AB_5_ toxins (Shiga toxin (Stx), subtilase cytotoxin (SubAB), cholera toxin (CT)) that comprise of a cell surface receptor-binding pentameric B-subunit are retrogradely trafficked via the Golgi apparatus to the ER where they trigger ER stress [9–11] (Fig. 2 (E)). The subunits of Stx1 are sensed as misfolded proteins within the ER and lead to UPR initiation [9, 12]. SubAB-triggered UPR activation is induced by the cleavage of BiP by this proteolytic toxin, resulting in subsequent loss of the inactivation process of the ER stress sensors [10]. CT, on the other hand, interacts directly with IRE1 leading to its activation [11].

## The role of individual ER stress marker activation

A closer look on the activation of the ER stress sensors illustrates that some bacterial factors induce all three axes of ER stress while others are more specific (Table 1). Examples of bacterial factors which trigger the activation of all three pathways include LPS, LLO, and SubAB [2, 5, 10], whereas *Chlamydia pneumoniae*, *S. pneumoniae*, *Legionella pneumophila*, and CT induce only one of the ER stress sensors [6, 11, 13, 14]. Considering that *S. pneumoniae* executes ER stress through a general response, that is through an increase in ROS, the activation of only the PERK-pathway observed might be due to suppression of IRE1- and ATF6-signaling pathway. Indeed, *L. pneumophila*, an intracellular pathogen which replicates within ER-like vacuoles, cleaves ATF6 but does not activate the PERK-signaling pathway, and it even blocks thapsigargin-induced *xbp1* splicing by expression of the two effectors Lgt1 and Lgt2 that glycosylate serine 53 in the elongation factor eEF1A causing protein synthesis inhibition [14]. Furthermore, ER stress sensor activation is cell-type specific as demonstrated in studies with Stx1 [9, 12]. Because a particular cell type is involved in a certain response within the host, this suggests that the activation of an individual ER stress marker may be cell-type specific and related to cell function.

**Table 1:** Bacteria and bacterial products that activate the ER stress sensors

Bacterium	Virulence factor	Cell type	UPR-specific host response			Mechanism	Reference
			IRE1	PERK	ATF6		
*Aeromonas hydrophila*	Aerolysin	HeLa	XBP1-s	n.d.	n.d.		[3]
*Bacillus thuringiensis*	Cry5B	*C. elegans*	*xbp1*-s	n.d.	n.d.	p38	[3]
*Brucella abortus*		BMM	*xbp1*-s	n.d.	n.d.		[16]
*Chlamydia pneumoniae*		HEp-2	–	eIF2α-p	–	Persistent infection	[13]
*Francisella tularensis*		BMM	*xbp1*-s	n.d.	n.d.	TLR2	[8]
Gram-negative bacteria	LPS	Monocytic THP-1	XBP1-s	PERK-p, eIF2α-p	ATF6 cleavage	TLR4	[2]
*Helicobacter pylori*	HP0175	AGS	n.d.	PERK-p, CHOP, ATF4, eIF2α-p	n.d.		[23]
*Legionella pneumophila*		BMM, HEK-293 FCγ, RAW264.7	Block of *xbp1-s*	Block of CHOP translation	ATF6 cleavage		[14]
*Listeria monocytogenes*	LLO	P388D1, HeLa	*xbp1*-s	eIF2α-p	ATF6 cleavage		[5]
*Mycobacterium tuberculosis*	ESAT-6	A549	*xbp1*-s	eIF2α-p, ATF4, *chop*	n.d.	ER Ca^2+^ release, ROS	[4]
*Pseudomonas aeruginosa*		*C. elegans*	*xbp1*-s	n.d.	n.d.	PMK-1 (p38 orthologue)	[7]
*Shigella dysenteriae*, STEC	Stx1	Monocytic THP-1	IRE1, *xbp1*-s	PERK-p, *chop*	ATF6 cleavage	Unfolded Stx (not for IRE1)	[9]
		Macrophage-like THP-1	IRE1-p, *xbp1*-s	PERK-p, CHOP	–	Unfolded Stx	[12]
*Staphylococcus aureus*		BMM, RAW264.7	*xbp1-s*	n.d.	n.d.	TLR2/4/9	[17]
STEC	SubAB	Vero, MEF	*xbp1*-s	*chop*, eIF2α-p, *atf4*	ATF6 cleavage	BiP cleavage	[10]
*Streptococcus pneumoniae*	H_2_O_2_	H441	decrease of *xbp1*-s	PERK-p, *atf4*, eIF2α-p, *atf3*, *chop*	–	ROS	[6]
*Streptomyces* sp.	Tunicamycin	P388D1, HeLa, *C*. e*legans*, J774	*xbp1*-s, IRE1-p	PERK-p, *chop*	ATF6 cleavage	Inhibition of N-linked glycosylation	[5, 7, 8]
*Vibrio cholerae*	CT	T84	IRE1-p, *xbp1*-s	–	–	Interaction with IRE1	[11]
*Yersinia pseudotuberculosis*		MEF, RAW264.7	n.d.	eIF2α-p, *atf3*	n.d.		[21]

### The role of IRE1 activation

Recent studies now show that, in addition to the transcriptional regulation of UPR targets, the IRE1-XBP1 arm also affects other host cell responses. Firstly, both IRE1 and XBP1 are implicated in cell-autonomous defense mechanisms against PFTs as *ire-1*- and *xbp-1*-negative *Caenorhabditis elegans* are more sensitive to Cry5B. In contrast, *pek-1* (a PERK homolog) did not contribute to resistance against Cry5B [3]. In addition, *xbp-1* but not *atf-6* or *pek-1* is required for the development and survival of *C. elegans* against *P. aeruginosa* [7]. Moreover, *xbp1*^*−/−*^ mice exhibit abnormalities within the intestine that include disseminated cellular ER stress, apoptosis of Paneth cells, and reduced expression of bactericidal molecules as well as mucin 2, thus permitting the dissemination of orally administered *Listeria monocytogenes* [15]. In addition, loss of *xbp1* increases the *Francisella tularensis* burden in the liver, spleen, and lung following aerosolic infection of mice. IRE1-triggered splicing of *xbp1* augments also the production of pro-inflammatory cytokines (interleukin 6 (IL-6)) in a TLR-dependent manner, as exemplified during *F. tularensis* infection [8]. Moreover, CT-activated IRE1 degrades mRNA associated with the ER membranes, a mechanism termed regulated IRE1-dependent decay (RIDD). The resulting mRNA fragments are sensed by retinoic acid inducible gene I (RIG-I) leading to production of cytokines like IL-6. IRE1α- and RIG-I-dependent expression of IL-6 is also observed after treatment of mouse embryonic fibroblasts with enzymatically inactive Stx suggesting a general mechanism of immune activation by AB_5_ toxins [11]. Recently, it was demonstrated that IRE1-extended ROS production stimulated caspase-2-Bid-mediated mitochondrial damage which activated the inflammasome [16]. Finally, *Staphylococcus aureus* induced IRE1-activation results in sustained generation of ROS which enables bacterial killing [17].

Secondly, in *Drosophila* S2 as well as in mammalian cells *ire1* but not *perk* and *atf6* is required for the replication of *Brucella melitensis*, a bacterium which grows in an ER-like compartment [18].

### The role of PERK activation

Translational attenuation allows a rapid spatial response to infection and contributes to host defenses through several mechanisms. Recently, it was described that PERK is involved in induction of the innate immune response which is triggered by the activation of the pro-inflammatory transcription factor NF-κB upon release of inhibitor of kappa B (IκB) [1]. Due to the short half-life of IκB, translational attenuation results in loss of NF-κB binding and subsequent expression of pro-inflammatory cytokines, as demonstrated during *L. pneumophila* infection [1, 19]. Moreover, PERK regulates the expression of the pro-apoptotic factor DNA-damage-inducible transcript 3 (*ddit3*, also known as *chop*) which contributes to host defense as shown during *Mycobacterium tuberculosis* infection where RNAi-mediated *chop* depletion results in an increased number of intracellular bacteria [20]. In addition, infection of cells defective in eIF2α phosphorylation results in a higher intracellular *L. monocytogenes*, *Yersinia pseudotuberculosis*, and *Chlamydia trachomatis* number [21]. On the other hand, phosphorylation of eIF2α is accompanied by increased expression of BiP which rescues host cells from stress contributing to host survival as observed during interferon gamma-induced persistent *C. pneumoniae* infection [13]. These studies suggest that translational attenuation serves as a mechanism of the host to detect bacteria and to activate an appropriate anti-bacterial defense.

A recent study showed that SubAB induces stress granules in various cells in a PERK-dependent manner [22]. Furthermore, Halder et al. reported that the *Helicobacter pylori* produced HP0175, a peptidyl-prolyl *cis-trans* isomerase, activates the PERK arm consequently leading to production of ATF4 and CHOP which both induce the expression of autophagy-related genes [23]. In contrast, SubAB-triggered PERK activation suppresses autophagy [24]. Thus, PERK-dependent autophagy induction seems to be insult dependent.

### The role of ATF6 activation

The role of bacterial-conditioned ATF6 activation is much less studied than that of IRE1 or PERK. Nonetheless, a recent study showed that *atf6*^*−/−*^ mice are highly susceptible to *Bacillus anthracis* infection and exhibit increased bacterial load in the spleens and livers. These effects are associated with reduced autophagic bacterial degradation as ATF6 was shown to be required for the expression of death-associated protein kinase 1 (*dapk1*) which promotes autophagy [25].

## Summary and future perspectives

It is now clear that host cells detect and respond to impairments of key cellular processes induced by microbial effectors rather than directly detecting the microbes themselves. These responses are generally accompanied by selective shutdown of essential cellular process such as protein biosynthesis. Indeed, it is remarkable that a large number of bacterial effector proteins, including toxins such as Stx, SubAB, or PFTs, target the eukaryotic translation machinery. UPR was described as a response of a cell to counteract the accumulation of the unfolded proteins within the ER. Surprisingly, during a bacterial infection, different ER stress sensors are activated indicating that bacteria have evolved strategies to induce a particular UPR pathway. Indeed, recent studies have revealed that the ER stress sensors modulate also other cell-autonomous processes such as autophagy, RIDD, the inflammasome, or stress granule formation. Chemical and pharmacological modulation of ER stress was shown to be essential for bacterial elimination. Interestingly, these compounds were not only used in cellular in vitro conditions but also in in vivo mouse experiments. Thus, these studies indicate that ER stress functions as a target in many pathologies, including acute infections and chronic neurodegenerative diseases. Therefore, targeting ER stress might be effective as an adjunct therapy in threatening bacterial infections.

## Abbreviations

ATF3/4/6: activating transcription factor 3/4/6
BiP: immunoglobulin heavy chain-binding protein
BMM: bone marrow-derived macrophages
CHOP: DNA-damage-inducible transcript 3
CT: cholera toxin
DAPK1: death-associated protein kinase 1
eIF2α: eukaryotic translation initiation factor 2α
ER: endoplasmic reticulum
ERAD: ER-associated degradation
ESAT-6: early secretory antigenic target 6
Grp94: heat shock protein 90 kDa beta member 1
IκB: inhibitor of kappa B
IL-6: interleukin 6
IRE1: inositol-requiring enzyme 1
LLO: listeriolysin O
LPS: lipopolysaccharide
MAPK: mitogen-activated protein kinase
mRNA: messenger RNA
PERK: protein kinase RNA (PKR)-like ER kinase
PFT: pore-forming toxin
RIDD: regulated IRE1-dependent decay
RIG-I: retinoic acid inducible gene I
ROS: reactive oxygen species
STEC: Shiga toxigenic *Escherichia coli*
Stx: Shiga toxin
SubAB: subtilase cytotoxin
TLR2/4/9: toll-like receptor 2/4/9
UPR: unfolded protein response
XBP1: X-box-binding protein 1

## References

[1] Chaudhari N, Talwar P, Parimisetty A, Lefebvre d‘Hellencourt C, Ravanan P (2014). A molecular web: endoplasmic reticulum stress, inflammation, and oxidative stress. Front Cell Neurosci.

[2] Coope A, Milanski M, Arruda AP, Ignacio-Souza LM, Saad MJ, Anhê GF, Velloso LA (2012). Chaperone insufficiency links TLR4 protein signaling to endoplasmic reticulum stress. J Biol Chem.

[3] Bischof LJ, Kao CY, Los FC, Gonzalez MR, Shen Z, Briggs SP, van der Goot FG, Aroian RV (2008). Activation of the unfolded protein response is required for defenses against bacterial pore-forming toxin in vivo. PLoS Pathog.

[4] Choi HH, Shin DM, Kang G, Kim KH, Park JB, Hur GM, Lee HM, Lim YJ, Park JK, Jo EK, Song CH (2010). Endoplasmic reticulum stress response is involved in *Mycobacterium tuberculosis* protein ESAT-6-mediated apoptosis. FEBS Lett.

[5] Pillich H, Loose M, Zimmer KP, Chakraborty T (2012). Activation of the unfolded protein response by *Listeria monocytogenes*. Cell Microbiol.

[6] Loose M, Hudel M, Zimmer KP, Garcia E, Hammerschmidt S, Lucas R, Chakraborty T, Pillich H (2015). Pneumococcal hydrogen peroxide-induced stress signaling regulates inflammatory genes. J Infect Dis.

[7] Richardson CE, Kooistra T, Kim DH (2010). An essential role for XBP-1 in host protection against immune activation in *C. elegans*. Nature.

[8] Martinon F, Chen X, Lee AH, Glimcher LH (2010). TLR activation of the transcription factor XBP1 regulates innate immune responses in macrophages. Nat Immunol.

[9] Lee SY, Lee MS, Cherla RP, Tesh VL (2008). Shiga toxin 1 induces apoptosis through the endoplasmic reticulum stress response in human monocytic cells. Cell Microbiol.

[10] Wolfson JJ, May KL, Thorpe CM, Jandhyala DM, Paton JC, Paton AW (2008). Subtilase cytotoxin activates PERK, IRE1 and ATF6 endoplasmic reticulum stress-signalling pathways. Cell Microbiol.

[11] Cho JA, Lee AH, Platzer B, Cross BC, Gardner BM, De Luca H, Luong P, Harding HP, Glimcher LH, Walter P, Fiebiger E, Ron D, Kagan JC, Lencer WI (2013). The unfolded protein response element IRE1α senses bacterial proteins invading the ER to activate RIG-I and innate immune signaling. Cell Host Microbe.

[12] Lee MS, Cherla RP, Leyva-Illades D, Tesh VL (2009). Bcl-2 regulates the onset of shiga toxin 1-induced apoptosis in THP-1 cells. Infect Immun.

[13] Shima K, Klinger M, Schütze S, Kaufhold I, Solbach W, Reiling N, Rupp J (2015). The role of endoplasmic reticulum-related BiP/GRP78 in interferon gamma-induced persistent *Chlamydia pneumoniae* infection. Cell Microbiol.

[14] Treacy-Abarca S, Mukherjee S (2015). *Legionella* suppresses the host unfolded protein response via multiple mechanisms. Nat Commun.

[15] Kaser A, Lee AH, Franke A, Glickman JN, Zeissig S, Tilg H, Nieuwenhuis EE, Higgins DE, Schreiber S, Glimcher LH, Blumberg RS (2008). XBP1 links ER stress to intestinal inflammation and confers genetic risk for human inflammatory bowel disease. Cell.

[16] Bronner DN, Abuaita BH, Chen X, Fitzgerald KA, Nunez G, He Y, Yin XM, O‘Riordan MX (2015). Endoplasmic reticulum stress activates the inflammasome via NLRP3- and caspase-2-driven mitochondrial damage. Immunity.

[17] Abuaita BH, Burkholder KM, Boles BR, O‘Riordan MX (2015). The endoplasmic reticulum stress sensor inositol-requiring enzyme 1α augments bacterial killing through sustained oxidant production. MBio.

[18] Qin QM, Pei J, Ancona V, Shaw BD, Ficht TA, de Figueiredo P (2008). RNAi screen of endoplasmic reticulum-associated host factors reveals a role for IRE1alpha in supporting *Brucella* replication. PLoS Pathog.

[19] Fontana MF, Banga S, Barry KC, Shen X, Tan Y, Luo ZQ, Vance RE (2011). Secreted bacterial effectors that inhibit host protein synthesis are critical for induction of the innate immune response to virulent *Legionella pneumophila*. PLoS Pathog.

[20] Lim YJ, Choi JA, Choi HH, Cho SN, Kim HJ, Jo EK, Park JK, Song CH (2011). Endoplasmic reticulum stress pathway-mediated apoptosis in macrophages contributes to the survival of *Mycobacterium tuberculosis*. PLoS One.

[21] Shrestha N, Bahnan W, Wiley DJ, Barber G, Fields KA, Schesser K (2012). Eukaryotic initiation factor 2 (eIF2) signaling regulates proinflammatory cytokine expression and bacterial invasion. J Biol Chem.

[22] Tsutsuki H, Yahiro K, Ogura K, Ichimura K, Iyoda S, Ohnishi M, Nagasawa S, Seto K, Moss J, Noda M (2016). Subtilase cytotoxin produced by locus of enterocyte effacement-negative shiga-toxigenic *Escherichia coli* induces stress granule formation. Cell Microbiol.

[23] Halder P, Datta C, Kumar R, Sharma AK, Basu J, Kundu M (2015). The secreted antigen, HP0175, of *Helicobacter pylori* links the unfolded protein response (UPR) to autophagy in gastric epithelial cells. Cell Microbiol.

[24] Yahiro K, Tsutsuki H, Ogura K, Nagasawa S, Moss J, Noda M (2014). DAP1, a negative regulator of autophagy, controls SubAB-mediated apoptosis and autophagy. Infect Immun.

[25] Gade P, Ramachandran G, Maachani UB, Rizzo MA, Okada T, Prywes R, Cross AS, Mori K, Kalvakolanu DV (2012). An IFN-γ-stimulated ATF6-C/EBP-β-signaling pathway critical for the expression of death associated protein kinase 1 and induction of autophagy. Proc Natl Acad Sci USA.

